# Genetic and antigenic characterization of two diarrhoeicdominant rotavirus A genotypes G3P[12] and G14P[12] circulating in the global equine population

**DOI:** 10.1099/jgv.0.002016

**Published:** 2024-08-20

**Authors:** Tirth Uprety, Shalini Soni, Chithra Sreenivasan, Ben M. Hause, Ahsan Naveed, Shuisong Ni, Amy J. Graves, Jennifer K. Morrow, Nathan Meade, Kenneth H. Mellits, Emma Adam, Michael A. Kennedy, Dan Wang, Feng Li

**Affiliations:** 1 Department of Veterinary Science, Maxwell H. Gluck Equine Research Center, University of Kentucky, Lexington, Kentucky 40546, USA; 2 Department of Veterinary and Biomedical Sciences, Animal Disease Research and Diagnostic Laboratory, South Dakota State University, Brookings, South Dakota, 57007, USA; 3 Department of Chemistry and Biochemistry, Miami University, Oxford, OH 45056, USA; 4 Equine Diagnostic Solutions, LLC, 1501 Bull Lea Rd, Suite 104, Lexington, Kentucky 40511, USA; 5 Division of Microbiology, Brewing, and Biotechnology, School of Biosciences, University of Nottingham, Sutton Bonington, UK

**Keywords:** equine rotavirus A, G3P[12], G14P[12], neutralization, phylogenetic analysis, virus isolation

## Abstract

Equine rotavirus species A (ERVA) G3P[12] and G14P[12] are two dominant genotypes that cause foal diarrhoea with a significant economic impact on the global equine industry. ERVA can also serve as a source of novel (equine-like) rotavirus species A (RVA) reassortants with zoonotic potential as those identified previously in 2013–2019 when equine G3-like RVA was responsible for worldwide outbreaks of severe gastroenteritis and hospitalizations in children. One hurdle to ERVA research is that the standard cell culture system optimized for human rotavirus replication is not efficient for isolating ERVA. Here, using an engineered cell line defective in antiviral innate immunity, we showed that both equine G3P[12] and G14P[12] strains can be rapidly isolated from diarrhoeic foals. The genome sequence analysis revealed that both G3P[12] and G14P[12] strains share the identical genotypic constellation except for VP7 and VP6 segments in which G3P[12] possessed VP7 of genotype G3 and VP6 of genotype I6 and G14P[12] had the combination of VP7 of genotype G14 and VP6 of genotype I2. Further characterization demonstrated that two ERVA genotypes have a limited cross-neutralization. The lack of an *in vitro* broad cross-protection between both genotypes supported the increased recent diarrhoea outbreaks due to equine G14P[12] in foals born to dams immunized with the inactivated monovalent equine G3P[12] vaccine. Finally, using the structural modelling approach, we provided the genetic basis of the antigenic divergence between ERVA G3P[12] and G14P[12] strains. The results of this study will provide a framework for further investigation of infection biology, pathogenesis and cross-protection of equine rotaviruses.

## Introduction

Rotavirus (RV) is the leading cause of diarrhoea-associated mortality in children that accounts for more than 19.11% of diarrhoea-associated deaths in the global paediatric population in 2019 [1]. RV is a triple layered, segmented, positive-sense double-stranded RNA virus that consists of 11 genomic segments coding for 6 structural and 6 nonstructural proteins [2]. The six structural proteins are VP1–VP4, VP6 and VP7, while non-structural proteins are NSP1–NSP5/6. RVs can be classified into nine different species, A–D and F–J, based on the antigenic differences and sequence homology of the middle-layer protein VP6 [3,5]. RV species A (RVA) [3] is the predominant species that causes infection and clinical diarrhoea in humans and animals across different age groups [2,6]. The outer glycoprotein VP7 (defining G genotype) and protease-cleaved VP4 (defining P genotype) form a dual nomenclature system (G and P genotyping) that has been used extensively for further classification of species A RVs [7]. The outer glycoprotein VP7 contains major neutralizing antigenic epitopes, while VP4 is considered the minor neutralizing antigen [8]. So far, 36 G and 51 P genotypes are reported from humans and animals [9].

In recent years, whole genome-based genotyping has been developed and harnessed for assigning the detailed genotypes for all 11 segments and has resulted in the contemporary genome constellation (GC) methodology that is gradually emerging as a tool to designate an RVA strain [3,10]. Using this approach, the genotypes of RVA are denoted as Gx-P[x]-Ix-Rx-Cx-Mx-Ax-Nx-Tx-Ex-Hx. RVA infections in humans are mainly caused by GCs I1-R1-C1-M1-A1-N1-T1-E1-H1 and I2-R2-C2-M2-A2-N2-T2-E2-H2 (barring G and P genotypes) that designate as Wa-like and DS-1 like [3]. Equine RVA has the GCs of I2/I6-R2-C2-M3-A10-N2-T3-E2/E12-H7 [11]. Similarly defined GCs have been demonstrated for RVA strains infecting bovine, porcine and canine. A partially shared GCs among different RVA strains may indicate that these viruses derived from the past reassortment events and have the potential in causing cross-species transmissions [3,12,14].

Although 36 G and 51 P genotypes have been identified in clinical cases in humans, G1, G2, G3, G4 or G9 in combination with P[8], and to a lesser extent, with P[4] or P[6], account for more than 90% of the global human RV infections [2]. RVA infection in foals was first reported in 1975 in the UK [15]. Since then, six G types (G3, G14, G5, G8, G10 and G13) and six P types (P[1], P[3], P[7], P[11], P[12] and P[18]) have been described in horses [16,25]. Although G3P[3], G3P[12], G14P[12], G5P[7], G8P[1], G10P[11] and G13P[18] are reported in foals, G3P[12] and G14P[12] are two predominant genotypes identified in the global equine population [26,32]. Equine G3 strains can be further classified into G3A and G3B based on five amino acid polymorphisms present in the three antigenic epitopes of VP7 and their reactivity to anti-equine RVA monoclonal antibodies [33]. Serological and epidemiological data indicate that two equine rotavirus A (ERVA) genotypes, G3P[12], and G14P[12], are ubiquitous and endemic in horse populations around the globe [22,27, 28, 33, 34].

ERVA-associated foal diarrhoea occurs predominantly in neonatal foals (less than 3 months of age) born to unvaccinated dams [16,32]. Prior to the development of the current monovalent ERVA vaccine, globally, ERVA-related diarrhoea in foals was shown to range from 20 to 77% of foal diarrhoea cases [35]. Clinically, the infection can manifest as life-threatening diarrhoea and is characterized by high morbidity [16,36], and thus, supportive care is necessary to overcome dehydration due to diarrhoea. Despite high morbidity, mortality is low due to supportive care that foals receive [16]. Mares are vaccinated against ERVA, and passive antibody transfer through colostrum protects foals during the initial months of life [29]. Mares are vaccinated at 8, 9 and 10 months of gestation with the inactivated ERVA vaccine containing G3P[12] strain H2 virus. This monovalent vaccine has been used extensively in horses in the USA, Europe, Australia and New Zealand and vastly reduced the incidence of neonatal diarrhoea. The equine industry in Japan has also used the similar vaccine format but with a different G3P[12] strain (HO-5), while horses in Argentina receive vaccination with an inactivated trivalent vaccine containing G3P[12] strain H2, G3P[2] strain SA11 and G6P[1] strain NCDV Lincoln [16,29].

These monovalent vaccines are mainly targeted against G3P[12] genotype; however, in recent years, G14P[12] has been reported to emerge as one predominant genotype in certain foaling seasons in foals between 3 and 5 months of age [27,37]. Studies from Japan suggest an alternating oscillating pattern of predominance between G3P[12] and G14P[12] strains [38,39]. While the monovalent G3P[12] vaccine, in general, has shown to be protective in decreasing the severity of clinical diarrhoea in foals [40,41], the experimental challenge studies conducted in equine farms with the monovalent G3P[12] vaccines have demonstrated that breakthrough infection with variants of concern of homologous G3P[12] genotype and heterologous G14P[12] strains can occur [42]. In the central Kentucky, outbreaks of ERVA-positive diarrhoea in foals 60–120 days of age where their dams have been immunized with an inactivated monovalent G3P[12] vaccine are still a concern for equine industry [35]. Both G3P[12] and G14 [12] strains were identified from affected foals, with some foals being infected with both strains. While clinical studies indicate some level of cross-protection by G3P[12] antibodies against G14P[12], the level of neutralization titres against the heterologous strain was much less compared with that against the homologous strain [43]. One complication for these clinical investigations is that previous exposure history of pregnant mares to various ERVA infections is unknown so the cross-neutralization between G3P[12] and G14P[12] could not be assessed precisely. This obstacle has shifted this research effort into small animal models where naive animals without previous exposure history to ERVA can be readily identified. For example, a suckling mouse model study showed that monovalent G3P[12] or G14P[12] vaccine provides protection against homologous virus challenge but has a limited protection against heterologous strains [44]. Despite the progress made, the extent of cross-neutralization in foals between two dominant genotypes, G3P[12] or G14P[12], remains poorly characterized. The other limitation for an in-depth antigenic property study of ERVA is a lack of contemporary G3P[12] and G14P[12] isolates. Sporadic isolation of ERVA G14P[12] strains has been reported from diseased foals in the USA, but G3P[12] strains have not been successfully isolated over the past four decades. Furthermore, the traditional virus isolation approach has been used extensively to isolate ERVA strains from clinical samples. This method includes 7–10 blind passage in African green monkey kidney epithelial cells (MA-104) or human colon carcinoma cells (Caco-2) [35]. Thus, some cell culture-adaptive changes in isolated strains may confer phenotypic differences in virus neutralization property when compared with clinical strains circulating in horses.

Due to the segmented nature of the rotaviral genome and genetic reassortment, ERVA can serve as a donor for the generation of novel equine-like RVA reassortants. These reassortants can infect and cause disease in humans, which has been demonstrated in 2013–2019 seasons where equine G3-like RVA was responsible for worldwide outbreaks of severe gastroenteritis and hospitalizations in children. On some occasions, unusual RVs, other than G3P[12] and G14P[12] genotypes, have been reported in equines. Equine RV G3P[3] is indicated to have originated from bats as an interspecies transmission event [30,45]. Thus, constant monitoring of circulating RV strains in equines is important from public health significance as well.

This study primarily focused on optimizing equine RV isolation protocol with minimal passaging by leveraging on an engineered cell line MA-104N*V cells defective in IFN and signal transducer activator of transcription 1 (STAT1) response. Rapid isolation of RVs of equine and other agricultural animals remains a challenge to veterinary research community. For example, in the USA, despite frequent faecal samples tested positive for equine RVA G3P[12] strain, over the past several decades, no G3 strains have been successfully isolated from diseased foals. We believed that this optimized isolation approach using engineered STAT and IFN defective cell line should be very useful for isolation and study of RVs of other groups/species. The other theme of this study was to generate equine RV genotype-specific rabbit antisera panel that could be used to characterize cross-reactivity between equine G3 and G14. Such a work has not been performed to date for the contemporary US equine RV isolates.

To better understand genetic and antigenic properties of ERVA, in this study, using an optimized virus isolation approach, we isolated both G3P[12] and G14P[12] strains of ERVA that are currently circulating in the US equine population, determined the full-genome sequences of these isolates and performed phylogenetic as well as structural analyses. Using rabbit-specific polyclonal antisera, we show that there is limited cross-neutralization between ERVA G3P[12] and G14P[12].

## Methods

### Cells, reagents and antibodies

African green monkey kidney epithelial cell line (MA-104) was grown in Dulbecco’s Modiﬁed Eagle Medium (DMEM) (Gibco, Invitrogen, USA) supplemented with 10% (vol/vol) FBS (Gibco, USA) and 100 U ml^−1^ penicillin–streptomycin (Life Technologies, Carlsbad, CA, USA). An African green monkey kidney epithelial cell line defective in STAT1 and IRF3 response (MA104 N*V) was obtained from the University of Nottingham. The cells were generated in-house at the University of Nottingham, and the detail description on generation of this cell line is provided in published article and dissertation [46,47]. MA104 N*V cells were propagated in DMEM supplemented with 10% FBS, 100 U ml^−1^ penicillin–streptomycin and 3 µg ml^−1^ each of puromycin (Gibco, A1113803) and blasticidin (Gibco, A1113903). Cells were cultured in the presence of 1 µg ml^−1^ plasmocin (InvivoGen, ant-mpp) to prevent mycoplasma contamination in cells. TPCK-Trypsin was purchased from Thermo Fisher (catalogue number: 20233). Goat-anti-RVA polyclonal antibody (catalogue number: PA1-7241), rabbit-anti-goat IgG Alexa Fluor 488 (catalogue number: A-11078) and goat anti-rabbit FITC (catalogue number: F-2765) were purchased from Thermo Fisher. Amphotericin B was purchased from Gibco (catalogue number: 15290018).

### Isolation of RV strains

RVA genotype G3P[12] or G14P[12] reverse transcription-PCR (RT-PCR)-positive faecal swabs in virus transport media were obtained from Equine Diagnostic Solutions (EDS LLC, Lexington, KY, USA). Samples with *C*_*t*_ values less than 20 were further processed for virus isolation. The faecal suspension was clarified by centrifugation at 3000 r.p.m. at 4 °C for 10 min. The supernatant thus obtained was filtered through a 0.45-µm filter. A volume of 500 µl of supernatant was incubated with 10 µg ml^−1^ of TPCK-Trypsin trypsin for 1 h at 37 °C. After an hour of incubation, serial tenfold dilutions of the trypsin-activated supernatant were prepared in plain media. Five hundred microlitres of undiluted and serial tenfold diluted (10^−1^, 10^−2^, 10^−3^ and 10^−4^) inoculum was inoculated into the confluent 6-well plates pre-seeded with MA-104N*V cells. Cells were washed with plain DMEM to remove any FBS before the addition of inoculum. The sixth well was used as negative control to visualize any differences in cell morphology between infected and uninfected cells. After 1 h of incubation at 37 °C, the inoculum was discarded, and 2 ml of DMEM containing 100 U ml^−1^ penicillin–streptomycin, 0.25 µg ml^−1^ of amphotericin B and 1 µg ml^−1^ of TPCK-trypsin were added to cells. Cells were incubated at 37 °C for 3–5 days and observed daily for the presence of cytopathic effect (CPE). The cells were freeze–thawed three times before further passaging into fresh monolayers of MA-104N*V cells. For passaging, the supernatant obtained after three freeze–thaw cycles was centrifuged at 3000 r.p.m. for 10 min at 4 °C. A volume of 500 µl of the cell debris-free supernatant was then activated by incubating in DMEM containing 0.5–1 µg ml^−1^ of TPCK trypsin followed by inoculation into fresh monolayers of MA-104N*V cells as mentioned earlier.

### Immunofluorescence assay

Media was discarded from virus-infected MA-104N*V cell monolayers, and cells were washed once with 1X PBS followed by fixation in 80% acetone. After fixation, cells were stained with primary (Goat-anti-RVA polyclonal antibody) and secondary (Rabbit-anti-goat IgG Alexa Fluor 488) antibody for 1 h each. Primary antibody was used at 1:200 dilution, while secondary antibody was used at 1:400 dilution. Cells were washed three times with 1X PBS before the addition of primary and secondary antibodies. Cells were counterstained with DAPI and visualized using Nikon Ti eclipse fluorescence microscope. Images were merged using Image J software (https://imagej.net).

### Semi-purification of RVs and negative stain electron microscopy

Equine RVs G3P[12] and G14[P12] were propagated in confluent monolayers of MA-104N*V cells and purified using previously reported protocol [48]. Briefly, after propagation, the culture flasks were freeze–thaw cycled three times. The cell pellet was removed from culture supernatant by centrifugation at 8000 r.p.m. for 20 min at 4 °C using a JA14 rotor in a Beckman J2-MC centrifuge. The clarified virus supernatant was transferred to fresh conical tube. Virus supernatants were then added to an ultracentrifuge tube (25×89 mm), with a 40% sucrose cushions prepared in TNC buffer (20 mM Tris-HCl; pH 8.0, 100 mM NaCl and 1 mM CaCl_2_). The supernatant was ultracentrifuged at 25 000 r.p.m. for 1.5 h at 4 °C using an SW32Ti rotor to pellet virus particles. The supernatant was decanted, and the pellet was resuspended in TNC buffer. Semi-purified viruses were analysed using negative stain electron microscopy (EM). Diluted semi-purified virus particles were placed on glow-discharged, carbon-coated EM grids for 1 min. Excess fluid was removed, and the grid was washed with ultrapure water. It was then negatively stained for 10 s using 1% uranyl acetate (pH 6.0) before finally examining under the electron microscope.

### Metagenomic sequencing, genotyping and phylogenetic analysis

Faecal sample and cell cultured virus were subjected to Illumina MiSeq-based metagenomics sequencing. Total RNAs were isolated using QIAamp viral RNA mini kit and kit protocol (Qiagen; catalogue number: 52904). Total RNAs thus obtained were subjected to Illumina MiSeq-based metagenomic sequencing. Depletion of ribosomal RNA, first- and second-strand cDNA synthesis, anchor ligation and library amplification were performed sequentially using Illumina Stranded Total RNA Prep and Ligation with Ribo-Zero Plus kit per kit protocol (Illumina, catalogue number: 20040525). Sequencing was performed using MiSeq Reagent kit V3 (Illumina, catalogue number: MS-102-3001). Adaptor trimmed raw reads were imported, followed by paired end read generation in CLC Genomics Workbench (Qiagen). *De novo* assembly of contigs was performed using the CLC Genomics, and the assembled contigs were subjected to blast analysis. Sequence obtained were submitted to GenBank under GenBank accession numbers: PP453575 to PP453596.

Genotyping of ERVA was performed using Virus Pathogen Resource (now Bacterial and Viral Bioinformatics Resource Center). Related RVA sequences from different species were obtained from Virus Variation Resource (https://www.ncbi.nlm.nih.gov/genome/viruses/variation/). Codon-based, nucleotide level multiple sequence alignment was performed in mega [49] with muscle, using default parameters in mega X. Phylogenetic analysis was performed in Mega X. The evolutionary history was inferred by using the maximum likelihood method with 1000 bootstrap replicates. Nucleotide substitution models used were general time reversible model (for VP1, VP2, VP3, VP4, VP6, NSP1 and NSP3), Tamura 92 model (for VP7), Tamura–Nei model (for NSP2), Hasegawa–Kishino–Yano model (for NSP4) and Tamura-3 parameter model (for NSP5). The initial trees for the heuristic search were automatically generated by applying the neighbour-join and BioNJ algorithms to a matrix of pairwise distances that were estimated using the maximum composite likelihood approach. The tree with the highest log likelihood value was then selected. Branch lengths were scaled to a size representing the number of nucleotide substitution per site. Phylogenetic trees were colour coded and annotated in iTOL:Interactive Tree of Life.

### Structure modelling and identification of the antigenic site differences in VP4 and VP7 proteins between ERVA G3P[12] and G14P[12]

The homology model of equine RVA VP7 was generated by homology modelling using MODELER based on the pairwise sequence alignment between equine RVA VP7 sequence and the simian VP7 sequence for which a crystal structure existed (PBD ID: 3FMG) using CLUSTALW. Antigenic sites in the outer glycoprotein VP7 were mapped based on previously reported studies [50]. The VP7 antigenic sites are divided into two defined epitopes: 7-1 and 7-2. The 7-1 epitope is further divided into 7-1a (with amino acid 87, 91, 94, 96–100, 104, 123, 125, 129, 130 and 291) and 7-1b (amino acid 201, 211–213, 238 and 242). The 7-2 antigenic epitope was formed by amino acid 143, 145–148, 190, 217, 221 and 264. Similarly antigenic sites in VP8* protein is divided into four predefined epitopes: 8-1 (aa 100, 146, 148, 150, 188, 190 and 192–196], 8-2 (aa 180 and 183), 8-3 (aa 113–116, 125, 131–133 and 135) and 8-4 (aa 87–89). Antigenic sites in VP5* are divided into five epitopes: 5-1 (aa 384, 386, 388, 393, 394, 398, 440 and 441), 5-2 (aa 434), 5-3 (aa 459), 5-4 (aa 429) and 5-5 (aa 306) [7,8, 51,55].

### Sequence logo

Site-specific amino acid conservation and difference were visualized using the sequence logo. For sequence logos, all available amino acid sequences of VP4 and VP7 for ERVA G3P[12] and ERVA G14P[12] genotypes were obtained from BV-BRC (https://www.bv-brc.org/). A total of 110, 260 and 209 partial or full-length coding sequences were used for generating sequence logos for P[12]-VP4, G3P[12]-VP7 and G14P[12]-VP7 proteins, respectively. These strains that derived these sequences for sequence logo analysis circulated between 1981 and 2021 in South America, Asia, Europe and North America. Sequences were first aligned in clustal omega (https://www.ebi.ac.uk/jdispatcher/msa/clustalo), and multiple sequenced aligned fasta was then used to generate sequence logos using WebLogo 3 [56] (https://weblogo.threeplusone.com/).

Each logo comprises stacks of letters (amino acids) with one stack representing each position in the analysed sequence. The sequence conservation at each position is indicated by the overall height of each stack (measured in bits). The height of symbols in each stack means the relative frequency of the corresponding amino acid at that position. The *X*-axis indicates the residue position in the antigenic sites (VP4 or VP7 protein), while the *Y*-axis showed the information content of occupying amino acid residue. The default colour scheme displaying different amino acids according to their different chemical properties is as follows: polar amino acids (G, S, T, Y, C, Q and N) coloured with green, basic (K, R and H) with blue, acidic (D and E) with red and hydrophobic (A, V, L, I, P, W, F and M) amino acids with black.

### Generation of ERVA genotype-specific rabbit reference sera and neutralization assay

ERVA G3P[12]- and G14P[12]-specific rabbit sera were generated by Covance (Labcorp). The representative strains of both genotypes, RVA/Horse-tc/USA/KY-0316FCL/2021/G14P[12] and RVA/Horse-tc/USA/KY-0809N/2021/G3P[12], were propagated in MA-104 cells. The supernatants were then purified by the ultracentrifugation, and the pellets were resuspended in 1X PBS. The titre of purified virus was 9×10^6^ focus forming unit (FFU) ml^−1^ and 7×10^6^ FFU ml^−1^ for G3P[12] and G14P[12], respectively. Both the viruses were inactivated simultaneously with 2 min brief UV exposure by placing the virus containing vials in biosafety cabinet with UV light on. Four ERVA sero-negative rabbits were immunized with G3P[12] and G14P[12] antigens, with *n*=2 for each antigen. The immunization regime involved one priming dose with Freund’s Complete Adjuvant (I/M) followed by three booster doses with Freund’s Incomplete Adjuvant (S/C). Terminal bleed rabbit serum was used for immunofluorescence assay (IFA) as well as for the neutralization assay.

For the neutralization assay, 300 FFU of trypsin-activated RVA/Horse-tc/USA/KY-0316FCL/2021/G14P[12] or RVA/Horse-tc/USA/KY-0809N/2021/G3P[12] virus was incubated with serial twofold dilutions of rabbit polyclonal serum to give a final volume of 100 µl. After 1 h of incubation, the virus–serum mix was inoculated into the confluent monolayer of MA-104 cells seeded in 96-well plates. After 1 h of incubation, the inoculum was discarded, and 100 µl of DMEM containing penicillin–streptomycin and 1 µg ml^−1^ of TPCK trypsin was added. After 18 h of incubation, the cell culture medium was discarded, and cells were fixed in 80% acetone for immunofluorescence staining. Fluorescence foci were determined by fluorescence focus forming assay as mentioned earlier. Neutralization titres were expressed as the reciprocal of the highest dilution that resulted in a 50% reduction in FFUs. Each assay was performed in duplicate, and each experiment was repeated three times.

### Statistical analysis

Statistical analyses were performed in GraphPad Prism version 9. To test statistical significance, multiple *t*-test with Welch’s correction was performed for viral replication kinetics data. Statistically significant data are indicated by an asterisk (*P<0.05; **P<0.01). Data are represented as mean±standard error of the mean (sem).

## Results

### Virus isolation, metagenomic sequencing and genotyping

**Fig. 1.**
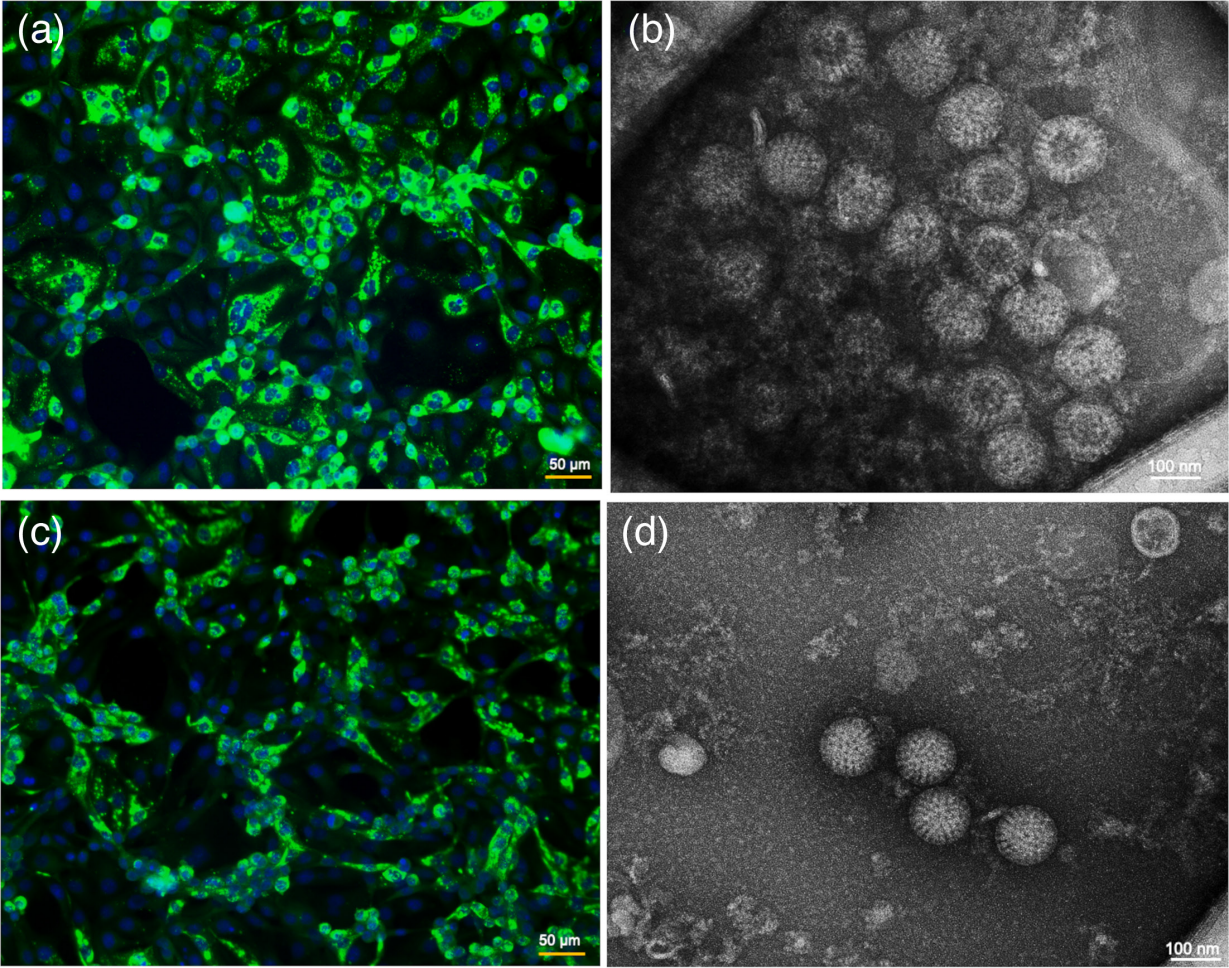
Isolation of two dominant genotypes of ERVA. Representative immunofluorescence imaging (IFA) of the cell monolayers inoculated with isolated virus supernatants shows the successful isolation of two currently circulating ERVA genotype G3P[12] (**a**) and G14P[12] (**b**). Electron microscopy of the pelleted culture supernatants from cells infected with ERVA G3P[12] (**c**) and G14P[12] (**d**), respectively, further confirmed isolated viruses to be rotavirus with the typical wheel-like icosahedral morphology. Figures are representative of three independent experiments. Scale bar for IFA images is 50 µm and for EM images is 100 nm.

Virus isolation was attempted from two ERVA G3P[12] and two G14P[12] RT-PCR assay-positive faecal swabs. These four faecal swabs originated from foals with clinical diarrhoea. The cell line used for ERVA isolation is an engineered MA-104 cell (MA-104 N*V) with a defect in STATI and IRF3 signalling pathways [47]. The engineered MA-104 N*V have been shown to enhance the RV replication efficiency in cell cultures as well as an increase in the rescue efficiency of reverse genetics system-derived RVs. By passage 2, CPE was observed for all dilutions of faecal suspensions. Immunofluorescence assay of MA-104 N*V-infected cells using supernatants derived from passage 2 cultures in combination with an RVA VP6-specific antibody demonstrated strong signals, indicating the replication of both G3P[12] (Fig. 1a) and G14P[12] (Fig. 1b) genotype strains with 100% successful isolation rate in this small-scale study. The successful isolation of both RVs was further confirmed by EM images showing characteristic RV wheel-like structure for both G3P[12] (Fig. 1c) and G14P[12] (Fig. 1d).

Using metagenomic sequencing, we have determined the full-genome sequences (except for partial non-coding regions) of passage two viruses (two G3 and two G14 isolates from cell culture) as well as corresponding faecal swabs that contained these strains. These sequences were aligned to identify any adaptive changes. While no amino acid differences were detected for ERVA G14P[12] strains between the cell culture-adapted viruses and faecal swabs, one amino acid difference (V344I) in the VP4 sequence was found between ERVA G3P[12] faecal swabs and corresponding cell-adapted viruses. The whole genome-based genotyping of two ERVA G3P[12] strains indicated that their genome constellation (GC) belongs to G3-P[12]-I6-R2-C2-M3-A10-N2-T3-E2-H7, while ERVA G14P[12] grouped with the GC cluster of G14-P[12]-I2-R2-C2-M3-A10-N2-T3-E2-H7. Blastp analysis (Table 1) of the 11 coding sequences of these showed high degree of similarity with existing ERVA G3P[12] or ERVA G14P[12].

**Table 1.** BLASTp analysis of the 11 proteins of RVA/Horse-tc/USA/KY 0809N/2021/G3P[12] and RVA/Horse-tc/USA/KY-0316FCL/2021/G14P[12]

Gene	Best blastp hit: Accession no.: Percentage identity(RVA/Horse-tc/USA/KY0809N/2021/G3P[12])	Best blastp hit: Accession no.: Percentage identity(RVA/Horse-tc/USA/KY-0316FCL/2021/G14P[12])
**VP1**	RVA/Horse-wt/ZAF/EqRV-SA1/2006/G14P[12]: AEX49913.1 : 99.54%	RVA/Horse-wt/ZAF/EqRV-SA1/2006/G14P[12] :AEX49913.1 : 99.72%
**VP2**	RVA/Horse-wt/ZAF/EqRV-SA1/2006/G14P[12]: AEX49914.1 : 99.09%	RVA/Horse-tc/JPN/HO-5/1982/G3P[12]: BAQ95486.1 : 100%
**VP3**	RVA/Horse-wt/ZAF/EqRV-SA1/2006/G14P[12]: AEX49915.1 : 99.28%	RVA/Horse-tc/JPN/HO-5/1982/G3P[12]: BAQ95487.1 : 99.64%
**VP4**	RVA/Horse-wt/ZAF/EqRV-SA1/2006/G14P[12]: AEX49916.1 : 98.58%	Equine Rotavirus A strain JE75: BAB40367.1 : 99.1%
**NSP1**	RVA/Horse-wt/ZAF/EqRV-SA1/2006/G14P[12]: AEX49917.1 : 98.37%	RVA/Horse-wt/JPN/No.59/2018/G14P12: BBI30133.1 : 99.59%
**VP6**	RVA/Horse-wt/JPN/No.101/2018/G3P12: BBI30107.1 : 100%	Equine Rotavirus A strain FI-23: Q86345.1 : 99.5%
**NSP2**	RVA/Horse-wt/IRL/04V2024/2004/G14P[12]: AEX60694.1 : 99.05%	Equine Rotavirus A strain H2: AIZ08933.1 : 99.68%
**NSP3**	RVA/Horse-tc/JPN/HH-22/1989/G3P[12]: AGS43836.1 : 97.76%	Equine Rotavirus A strain FI23: AIZ08945.1 : 98.71%
**VP7**	Equine Rotavirus A strain 4775G7: AAV28226.1 : 99.69%	Equine Rotavirus A strain FI-23: AIZ08942.1 : 99.69%
**NSP4**	RVA/Horse-wt/ZAF/EqRV-SA1/2006/G14P[12]: AEX49922.1 : 98.86%	RVA/Horse-wt/JPN/No.59/2018/G14P12: BBI30136.1 : 98.86%
**NSP5**	RVA/Horse-wt/IRL/03V04954/2003/G3P[12]: AEX60689.1 : 98.99%	RVA/Horse-wt/JPN/No.59/2018/G14P12: BBI30137.1 : 100%

### Phylogenetic analysis

**Fig. 2.**
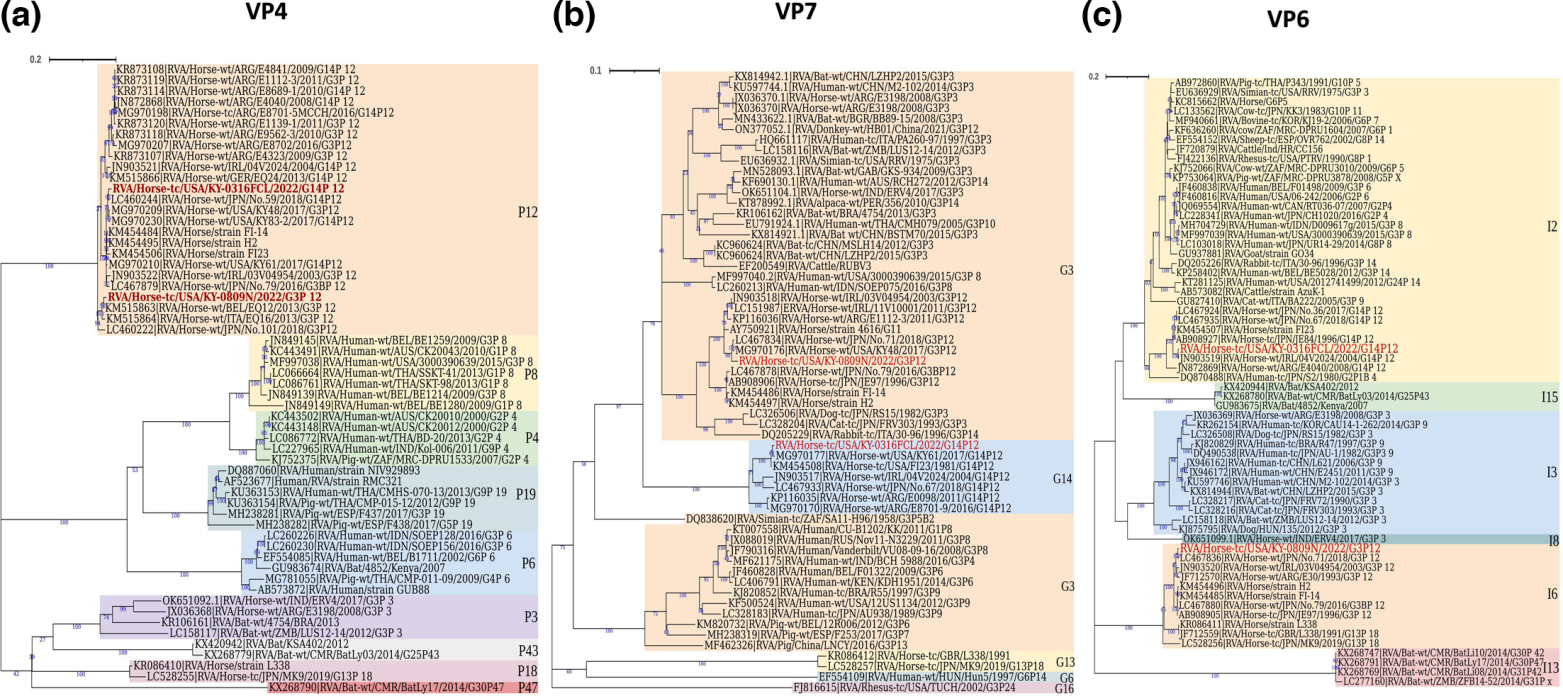
Phylogenetic analyses of VP4, VP7 and VP6 protein sequences of isolated ERVA G3P[12] and G14P[12]. Phylogenetic tree analyses for VP4 (**a**), VP7 (**b**) and VP6 (**c**) protein sequences were inferred by using the maximum likelihood method with 1000 bootstrap replicates. Bootstrap values are shown in blue. The tree with the highest log likelihood is shown. The tree is drawn to scale, with branch lengths measured in the number of substitutions per site. Evolutionary analyses were conducted in mega X. General time reversible model of nucleotide substitution was used for VP4 and VP6, while Tamura 92 model was used for VP7. The phylogenetic trees were colour coded and annotated in iTOL:Interactive Tree of Life.

To further understand viral evolution and genetic relatedness with other species A RVs of equine and other animal species, we performed the phylogenetic analysis on both ERVA G3P[12] and ERVA G14P[12] strains isolated in this study. Nucleotide-based phylogenetic analysis of the coding sequences of VP4, VP7 and VP6 are shown in Fig. 2a, b and c, respectively. The VP4 spike protein (defining P genotype) is cleaved into two subunits, VP8* and VP5*, with VP8* involved in binding the cell surface glycan receptor and VP5* responsible for the membrane penetration [57]. Both VP8* and VP5* proteins are targets of virus-neutralizing antibodies. The VP4-based phylogenetic analysis indicated that RVA/Horse-tc/USA/KY-0316FCL/2021/G14P[12] and RVA/Horse-tc/USA/KY-0809N/2021/G3P[12] were distantly related despite their clustering together within the P[12] genotype. Interestingly, our ERVA G3P[12] strain was more related to a previously reported ERVA G14P[12] strain isolated from central Kentucky in 2017 than our ERVA G14P[12] strain (Fig. 2a), suggesting a distinct evolutionary pathway of the VP4 protein in these contemporary ERVA G3P[12] and G14P[12] strains isolated in this study.

The function of VP7 protein remains poorly understood, but roles in viral uncoating, genome transcription and viral assembly have been proposed [2,8, 51]. VP7 protein is thought to be a primary target of the virus-neutralizing antibody response [58]. The VP7-based phylogenetic analysis showed that RVA/Horse-tc/USA/KY-0809N/2021/G3P[12] is more closely related to equine-like human G3 as well as canine G3 (Fig. 2b). RVA/Horse-tc/USA/KY-0316FCL/2021 /G14P[12] grouped together with other equine G14 strains. Interestingly, equine G14 and G3 genotypes seem to emerge from a common ancestor. While equine G3P[3] from India and Argentina clustered together with bat RVA G3P[3], RVA/Donkey-wt/HB01/China/2021/G3P[12] shared a common ancestor with RVA/Bat-wt/BGR/BB89-15/2008 /G3P[3] (Fig. 2b). Interestingly, bat RVA/G3P[3] were closely related to human G3P[3], G3P[10] and G3P[14], in addition to being highly associated with equine G3P[3] and RVA/Donkey-wt/HB01/China/2021 /G3P[12] (Fig. 2b).

VP6 protein consists of the middle-layer lattice of an RVA particle. Several studies have shown that antibodies targeting the conserved VP6 protein are protective against RVA infection in cell cultures and animal models [59]. The VP6-based phylogenetic analysis indicated that RVA/Horse-tc/USA/KY-0809N/2021/G3P[12] clustered together with ERVA G3P[12] from Argentina, Japan and Ireland, along with ERVA G13P[18], which all belong to I6 genotype (Fig. 2c). Interestingly, RVA/Horse-tc/USA/KY-0316FCL/2021/G14P[12], along with ERVA G6P[5], and equine-like human G3P[8] clustered into the I2 genotype (Fig. 2c). Further examination demonstrated that ERVA G14P[12] strains belonging to I2 genotype shared a common ancestor with bat RVA belonging to the I15 genotype. Two ERVA G3P[3] strains, one from Argentina and another from India, are thought to originate from bats [30,45, 60]. VP6 segment of the ERVA G3P[3] strain from Argentina was phylogenetically closer to those of human and bat RVA strains consisting of the I3 genotype, while ERVA G3P[3] from India clustered separately as a monophyletic clade into the I8 genotype (Fig. 2c).

**Fig. 3.**
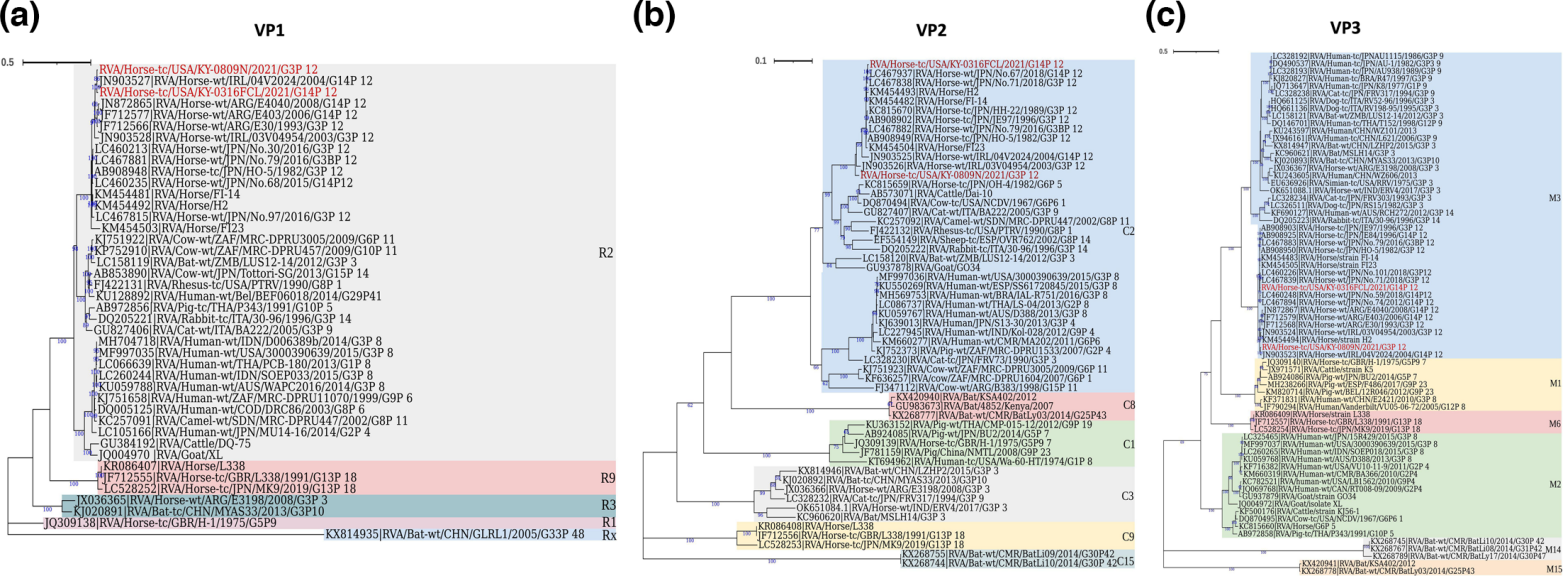
Phylogenetic analyses of VP1, VP2 and VP3 protein sequences of isolated ERVA G3P[12] and G14P[12]. Phylogenetic tree analyses for VP1 (**a**), VP2 (**b**) and VP3 (**c**) protein sequences were inferred by using the maximum likelihood method with 1000 bootstrap replicates. Bootstrap values are shown in blue. The tree with the highest log likelihood is shown. The tree is drawn to scale, with branch lengths measured in the number of substitutions per site. Evolutionary analyses were conducted in mega X. General time reversible model of nucleotide substitution was used for VP1, VP2 and VP3. The phylogenetic trees were colour coded and annotated in iTOL:Interactive Tree of Life.

VP1 protein is the largest protein expressed by RVAs. It acts as the RNA-dependent RNA polymerase to replicate viral RNA genome [61]. VP1-based phylogenetic analysis showed that both RVA/Horse-tc/USA/KY-0316FCL/2021/G14P[12] and RVA/Horse-tc/USA/KY-0809N/2021/G3P[12] clustered together with other ERVA G3P[12] and G14P[12] strains previously identified (Fig. 3a). All equine G3P[12] and G14P[12] VP1 segments were originated from a common node that was classified into the R2 genotype. The R2 genotype also includes RVAs of humans and other species such as cattle and bats. These RVAs appeared to share a common ancestor with equine viruses (Fig. 3a). Nevertheless, VP1 sequences of three equine RVA strains, G13P[18] (R9 genotype), ERVA G5P[9] (R1 genotype) and ERVA G3P[3] (R3), formed distinct phylogenetic clades from that formed by G3P[12] and G14P[12] strains (Fig. 3a). ERVA G3P[3] from Argentina clustered together with bat RVA G3P[10] from China (Fig. 3a) on VP1-based phylogenetic analysis.

We also conducted phylogenetic analyses for VP2 and VP3 segments of ERVA G3P[12] and G14P[12] strains. VP2 is the inner capsid protein layer, while VP3 provides a capping enzymatic function for RVA genome replication [51]. VP2-based phylogenetic analysis showed that both RVA/Horse-tc/USA/KY-0316FCL/2021/G14P[12] and RVA/Horse-tc/USA/KY-0809N/2021/G3P[12] clustered in C2 genotype, along with other ERVA G3P[12] and G14P[12] strains (Fig. 3b). Human RVAs and ERVA G6P[5] strain from Japan were also clustered in C2 genotype. It is intriguing to note that ERVA G5P9[7] was more closely related to porcine G5P[7] and porcine G9P[23], all of which clustered in C1 genotype. Similarly, ERVA G3P[3] from both India and Argentina clustered together with bat RVA G3P[3], belonging to the C4 genotype, while ERVA G13P[18] formed a monophyletic clade that was clustered in the C9 genotype (Fig. 3b). On VP3-based phylogenetic analysis, both RVA/Horse-tc/USA/KY-0316FCL/2021/G14P[12] and RVA/Horse-tc/USA/KY-0809N/2021/G3P[12] clustered together, grouping into the M3 genotype, while ERVA G3P[3] strains from Argentina and India, despite belonging to the M3 genotype, clustered separately and were more closely related to simian RVA G3P[3], bat RVA G3P[3] and G3P[10] (Fig. 3c). Human RVAs were also classified into the M3 genotype. Interestingly, some equine RVAs diverged from equine G3P[12] and G14P[12] strains in VP3 gene evolutionary pathway. For example, ERVA G5P9[7] clustered together with cattle strain K5 and porcine RVA G5P[7] strain under the M1 genotype (Fig. 3c), while ERVA G13P[18] formed a distinct phylogenetic cluster (M6 genotype). Interestingly, equine-like G3P[8] showed a close common ancestral relationship to ERVA G6P[5] as both of them clustered in the M2 genotype (Fig. 3c).

**Fig. 4.**
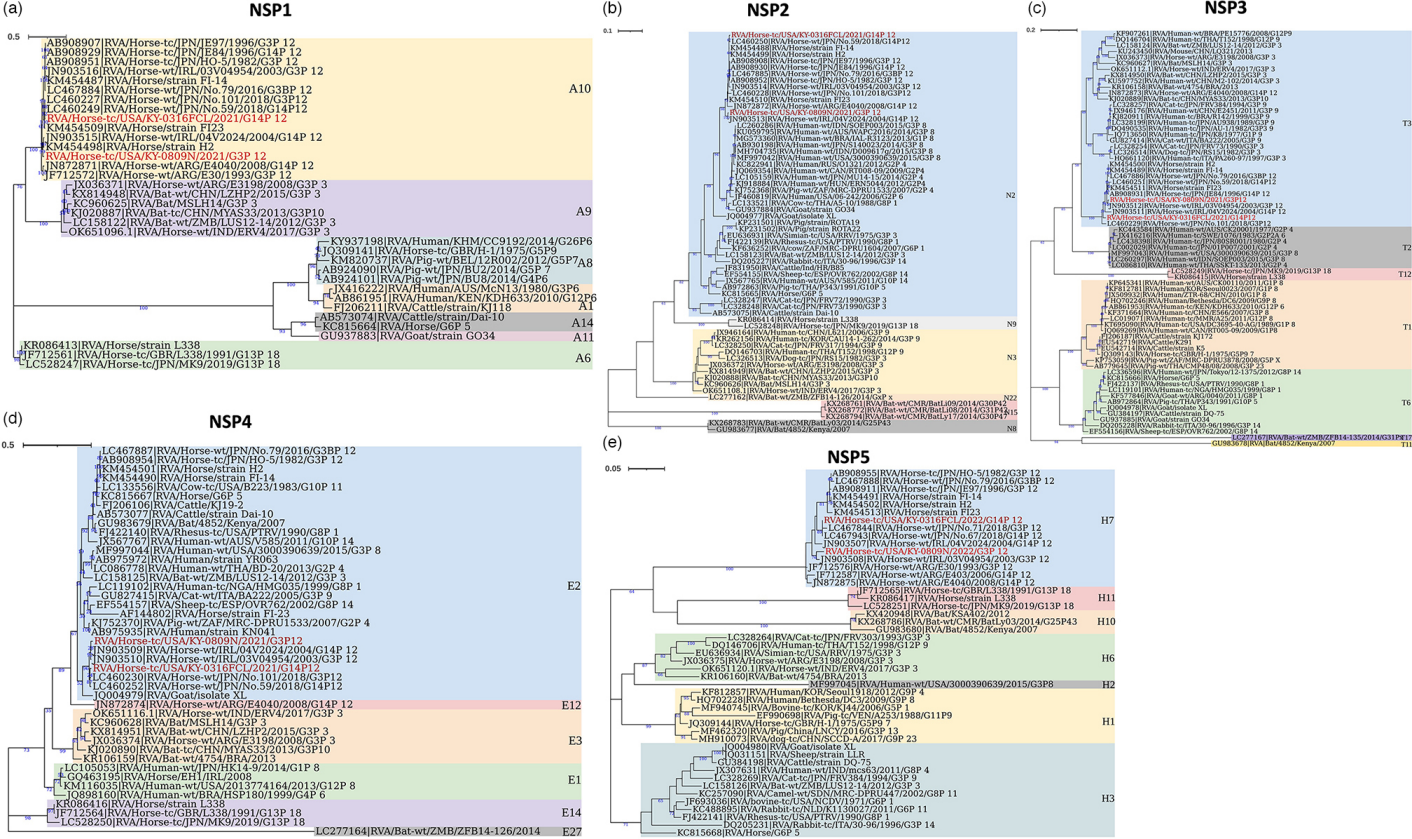
Phylogenetic analyses of non-structural protein sequences of isolated ERVA G3P[12] and G14P[12]. Phylogenetic tree analyses for non-structural protein sequences, NSP1 (**a**), NSP2 (**b**), NSP3 (**c**), NSP4 (**d**) and NSP5 (**e**), were inferred by using the maximum likelihood method with 1000 bootstrap replicates. Bootstrap values are shown in blue. The tree with the highest log likelihood is shown. The tree is drawn to scale, with branch lengths measured in the number of substitutions per site. Evolutionary analyses were conducted in mega X. General time reversible model of nucleotide substitution was used for NSP1 and NSP3, Tamura–Nei model was used for NSP2, Hasegawa–Kishino–Yano model was used for NSP4 and Tamura-3 parameter model was used for NSP5. The phylogenetic trees were colour coded and annotated in iTOL:Interactive Tree of Life.

Non-structural protein 1 (NSP1)-based phylogenetic analysis indicated that, while RVA/Horse-tc/USA/KY-0316FCL/2021/G14P[12] was closely related to ERVA G14P[12] from Japan, the RVA/Horse-tc/USA/KY-0809N/2021/G3P[12] was more closely related to ERVA G14P[12] from Argentina (Fig. 4a) despite both G3P[12] and G14P[12] belonging to the A10 genotype. ERVA G3P[3] clustered together with bat ERVA G3P[3] and G3P[10], forming a separate phylogenetic clade that belongs to the A9 genotype (Fig. 4a). While ERVA G5P[9] was more closely related to porcine RVA belonging to the A8 genotype, ERVA G6P[5] was more closely related to cattle RVA strain Dai-10 (A14 genotype), and ERVA G13P[18] formed a distinct cluster and was more distantly related to ERVA G3P[12] and G14P[12] (Fig. 4a). Phylogenetic tree analysis of NSP2 showed that both contemporary RVA/Horse-tc/USA/KY-0316FCL/2021/G14P[12] and RVA/Horse-tc/USA/KY-0809N/2021 /G3P[12] were more closely related to past ERVA G14P[12] strains than to circulated ERVA G3P[12] strains (Fig. 4b), despite being clustered into the N2 genotype. While ERVA G6P[5] was also grouped into the N2 genotype, it was distantly related to ERVA G3P[12] and ERVA G14P[12] strains (Fig. 4b). ERVA G13P[18] clustered separately into the N9 genotype, while ERVA G3P[3] and bat ERVA G3P[3] and G3P[10] were closely related, belonging to the N3 genotype (Fig. 4b).

In NSP3-based phylogenetic analysis, both RVA/Horse-tc/USA/KY-0316FCL/2021/G14P[12] and RVA/Horse-tc/USA/KY-0809N/2021/G3P[12], were closely related, which were clustered into the T3 genotype (Fig. 4c). While ERVA G3P[3] isolated in Argentina clustered together with mouse RVA from China with bat RVA G3P[3] as the nearest common ancestor (Fig. 4c), ERVA G3P[3] from India showed a close phylogenetic association with bat RVA G3P[3] (Fig. 4c). Interestingly, a previously circulated RVA/Horse-wt/ARG/E4040/2008/G14P[12] was closely related to RVA/Bat-wt/4754/BRA/2013 and bat RVA G3P[10] from China. Furthermore, ERVA G13P[18], G5P9[7] and G6P[5] clustered separately into T12, T1 and T6 genotypes, respectively (Fig. 4c). Phylogenetic tree analyses of NSP4 and NSP5 segments demonstrated that RVA/Horse-tc/USA/KY-0316FCL/2021/G14P[12] and RVA/Horse-tc/USA/KY-0809N/2021/G3P[12] clustered into a common phylogenetic clade (Fig. 4d, and Fig. 4e, respectively). Consistent with other segments, ERVA G3P[3] from Argentina and India showed a close phylogenetic association with bat RVA G3P[3] in both NSP4- and NSP5-based phylogenetic analyses (Fig. 4d and e), while ERVA G13P[18] clustered separately in both segments. One particular standout strain was that previously isolated RVA/Horse-wt/ARG/E4040/2008/G14P[12] diverged substantially from most ERVA G3P[12] and G14P[12] strains (the E2 genotype) in NSP4-based phylogenetic analysis, belonging to the E12 genotype (Fig. 4d). The E2 genotype also include RVAs of human and other species such as cattle and goat.

### Serological cross-reactivity and cross-neutralization between ERVA G3P[12] and ERVA G14P[12]

**Fig. 5.**
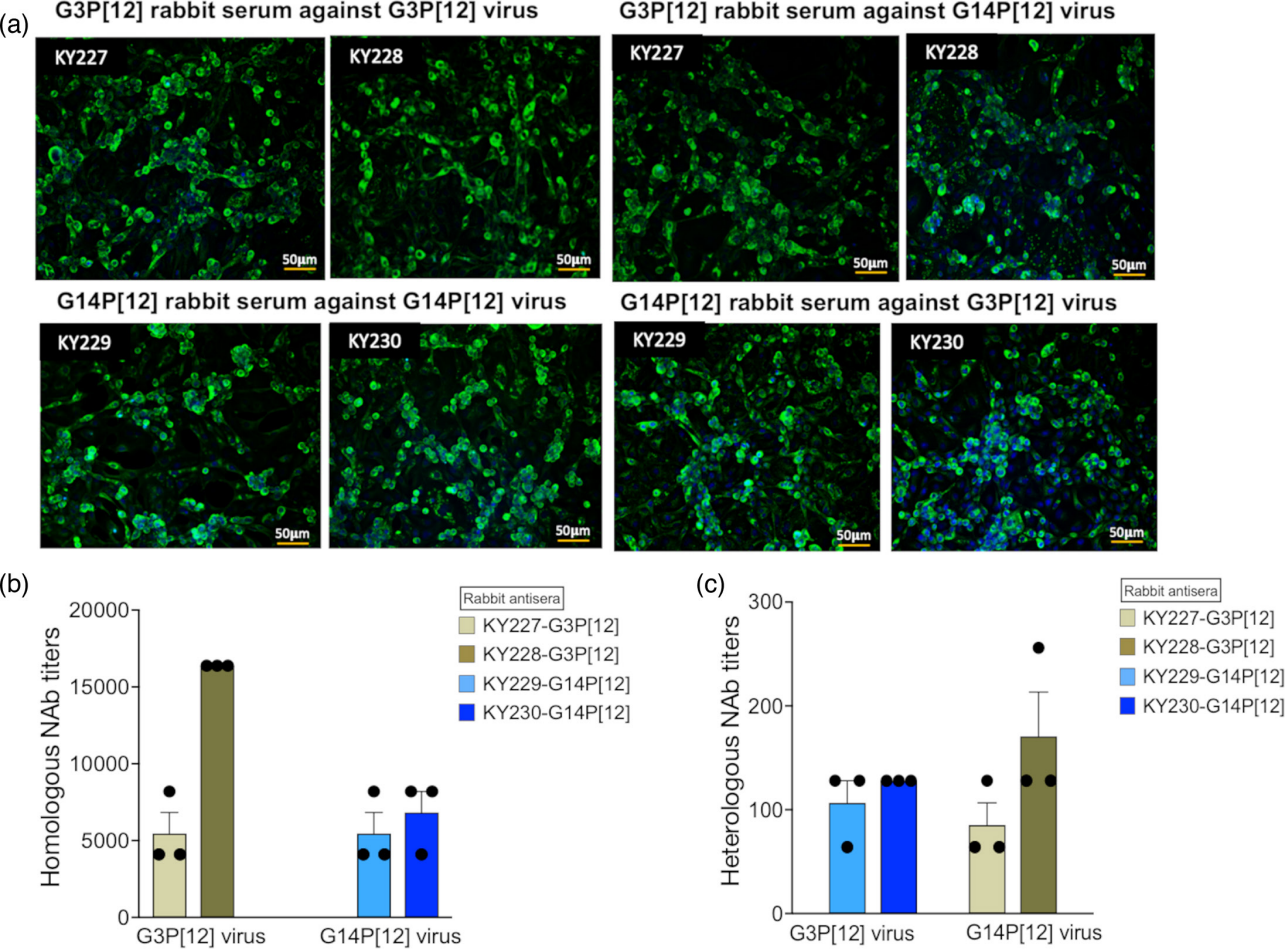
Serological cross-reactivity between ERVA G3P[12] and G14P[12]. Serological cross reactivity between ERVA G3P[12] and G14P[12] was assessed by generating virus-specific rabbit polyclonal sera followed by the neutralization assay. Four rabbits, two each for G3P[12] and G14P[12], were immunized by the purified UV inactivated virus preparation. G3P[12]- and G14P[12]-specific terminal polyclonal sera recognized both G3P[12] and G14P[12] viruses in the IFA (**a**). Homologous and heterologous neutralization titres of virus-specific rabbit sera were measured using standard rotavirus neutralization assay (**b, c**). IFA images are representative of three independent experiments. Scale bar represents 50 µm in panel a. Neutralization titres represent the mean of three independent experiment, and error bar represents standard error of mean.

Serological cross-reactivity and cross-protective neutralization activity between RVA/Horse-tc/USA/KY-0316FCL/2021/G14P[12] and RVA/Horse-tc/USA/KY-0809N/2021/G3P[12] were assessed by virus-specific rabbit antisera, immunofluorescence assay and virus-neutralization assay. Two rabbit anti-RVA/Horse-tc/USA/KY-0809N/2021/G3P[12] sera were labelled as KY227 and KY228, while two rabbit anti-RVA/Horse-tc/USA/KY-0316FCL/2021/G14P[12] sera were named as KY229 and KY230. Results of immunofluorescence assay revealed that viral antigens were detected by both homologous and heterologous sera, regardless of the viral genotype used for infection of MA-104 cells (Fig. 5a).

To characterize the virus neutralization properties of RVA/Horse-tc/USA/KY-0316FCL/2021/G14P[12] and RVA/Horse-tc/USA/KY-0809N/2021/G3P[12], we performed the standard fluorescent focus neutralization assay with MA-104 target cells using the rabbit reference antisera raised against purified virus particle preparations of these two strains. In the virus neutralization assay, ERVA G3P[12] rabbit sera KY227 and KY228 had geometric mean virus neutralization antibody titres (GMT) of 5160 and 16 384, respectively, against the homologous G3P[12] (Fig. 5b). Similarly, ERVA G14 rabbit sera KY229 and KY230 had geometric mean titres (GMT) of 5160 and 6501, respectively, against the homologous RVA/Horse-tc/USA/KY-0316FCL/2021/G14P[12] (Fig. 5b). Two-tailed *t*-test indicated no statistical difference between homologous titres (data not shown). In a marked contrast, the two anti-G3P[12] rabbit sera did not efficiently recognize and neutralize heterologous G14P[12], with a titre 64- or 128-fold lower than the homologous titre, which was also true for the two anti-G14P[12] rabbit sera in neutralizing heterologous G3 virus (Fig. 5c). These results clearly indicate a limited cross-neutralization of ERVA G3P[12] and G14P[12] isolates in the context of the rabbit antisera raised against the purified virus particles. However, no statistical difference was observed in homologous neutralization titres, paired-two tailed *t*-test for homologous and heterologous titre of G3P[12]-specific rabbit sera, and G14P[12]-specific rabbit antisera showed statistical differences in titres (*P*<0.0075 and *P*<0.0011).

### Structure modelling for the identification of the antigenic-site differences between ERVA G3P[12] and G14P[12]

To provide structural insights into the observed difference in virus neutralization properties between two dominant ERVA genotypes circulating in horses, we mapped the antigenic determinants onto structural models of the VP8* domain of VP4 and the VP7 protein, primary targets of virus neutralization antibodies.

**Fig. 6.**
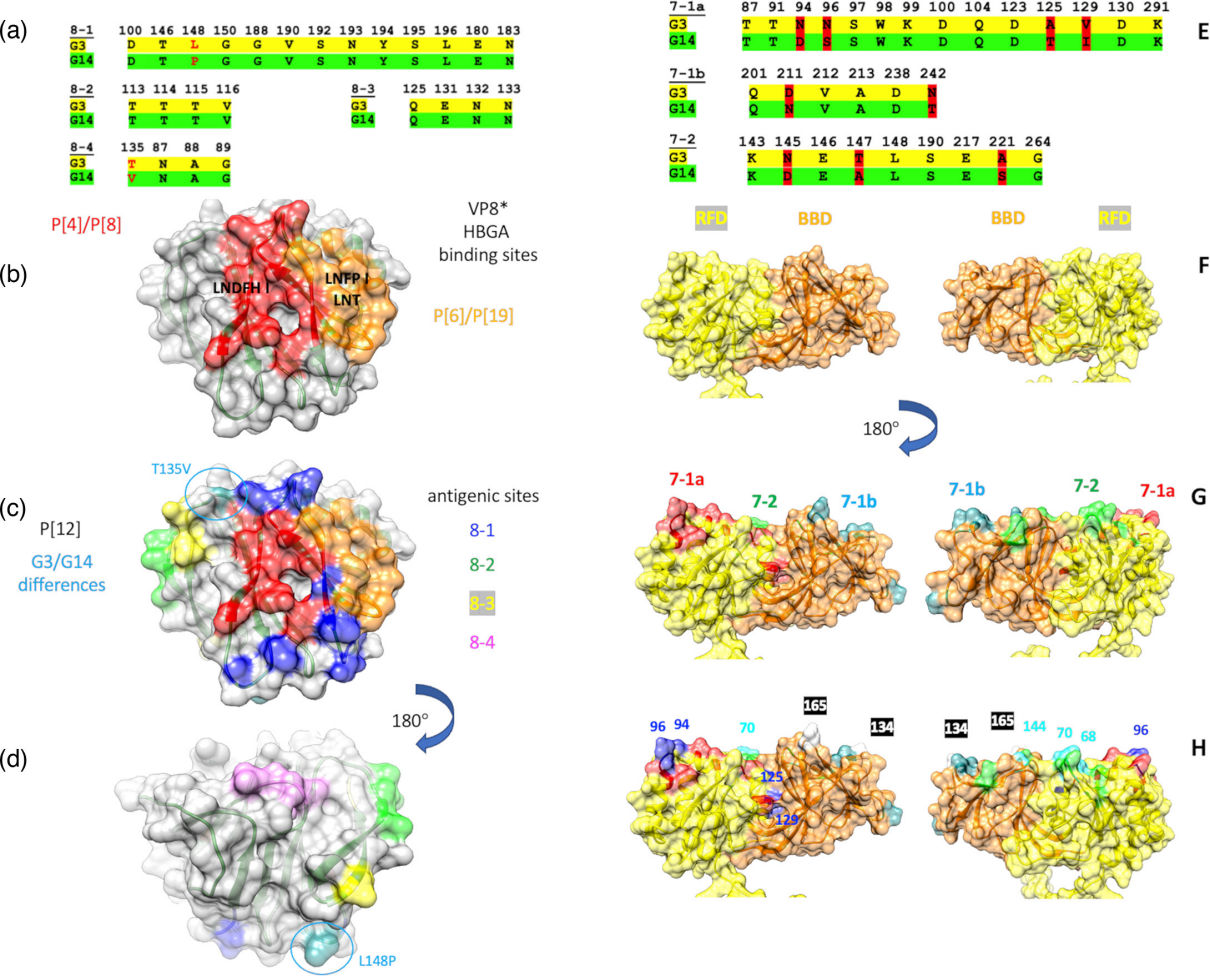
Antigenic differences between ERVA G3P[12] and G14P[12] in VP8* and VP7 protein. Pairwise sequence alignment obtained in ClustalW was used to generate the homology model using MODELER within the Chimera software package. (**a**) The three antigenic epitopes in VP8*, including mutations between G3P[12] and G14P[12], are shown. The antigenic epitopes of equine P[12] were mapped to the currently known receptor-binding sites of the VP8* receptor-binding domains for the four human P genotypes in the P[I] genogroup, i.e. P[4], P[6], P[8] and P[19] (**b**). The differences in amino acid residues between equine G3P[12] and G14P[12] in the VP8* receptor-binding domain are highlighted across four known antigenic sites in VP8* (**c**). The protein structure was rotated 180^o^ to show both sides of protein (**d**). (**e**) The three antigenic epitopes in VP7 7-1 a, 7-1b and 7-2 are shown. The homology model of ERVA VP7 was generated by homology modelling using MODELER based on the pairwise sequence alignment between the ERVA VP7 sequence and the simian VP7 sequence (**f**). The RFD is coloured yellow, and the BBD is coloured orange. The structure is rotated by 180^o^ to show both sides of the protein in (**f, g and h**). The three antigenic epitopes in VP7 are colour coded in red (7-1a), teal (7-1b) and green (7-2), which are mapped onto the model (**g**). The amino acid differences in the VP7 antigenic sites between the G3 and G14 genotypes are mapped in (**h**) relative to the epitope sites colour coded as in (**g**).

The VP4 protein was highly conserved (97.9% homology) between ERVA G3P[12] and G14P[12] genotypes. Of the 17 amino acid mutations, only two occurred in the antigenic epitope region of VP8*, while no antigenic mismatches were observed in the antigenic epitope region of VP5*. The L148P mutation in the VP8* domain occurred in the antigenic epitope 8-1, while mutation T135V was in the antigenic epitope 8-4 (Fig. 6a). Mapping all 17 mutations onto a homology model of the P[12] VP8* receptor-binding domain based on the X-ray crystal structure of the human P[8] VP8* receptor-binding domain (PDB ID=6 VKX) [62] revealed the positional relationship of the mutations in the antigenic epitopes relative to the known ββ and βα sites for binding histo-blood group antigens (HBGAs) (Fig. 6b). In Fig. 6c, the antigenic sites are mapped onto the receptor-binding domain to indicate their locations relative to the ββ- and βα-binding sites. The T135V mutation is found to be adjacent to the ββ-binding site (Fig. 6c), whereas the L148P mutation can be seen to be distant (on the opposite side of the protein) from both known receptor-binding sites (Fig. 6d). Although not in antigenic site, three amino acid differences (I136T, K145R and S151N) between G14P[12] and G3P[12] VP8* were identified near the antigenic sites. We found an overall 88% homology of VP7 protein at the amino acid sequence level between RVA/Horse-tc/USA/KY-0809N/2021/G3P[12] and RVA/Horse-tc/USA/KY-0316FCL/2021/G14P[12]. Of the 39 amino acid differences, nine amino acid residues were located in the antigenic epitopes of VP7 (Fig. 6e). Antigenic residue mismatches between ERVA G3P[12] and G14P[12] in antigenic epitope 7-1a were N94D, N96S, A125T and V129I, D211N and N242T in antigenic epitope 7-1b and N145D, T147A and A221S in antigenic epitope 7-2 (Fig. 6e). The antigenic sites and mutations were mapped onto a homology model of simian (SA11) RVAVP7 (PDB ID=3 FMG) [8], which contains both a Rossmann fold domain (RFD) and a β-barrel domain (BBD) (Fig. 6f). The 7-1a, 7-1b and 7-2 antigenic epitopes were mapped onto the homology model, as depicted in Fig. 6g. The mutations in the antigenic epitopes are indicated with respect to their respective antigenic epitopes in Fig. 6h. Collectively, the mapping indicates that mutations between the G3P[12] and G14P[12] antigenic epitopes are broadly distributed spanning both the RFD and BBD, which occur in all three identified antigenic epitopes.

**Fig. 7.**
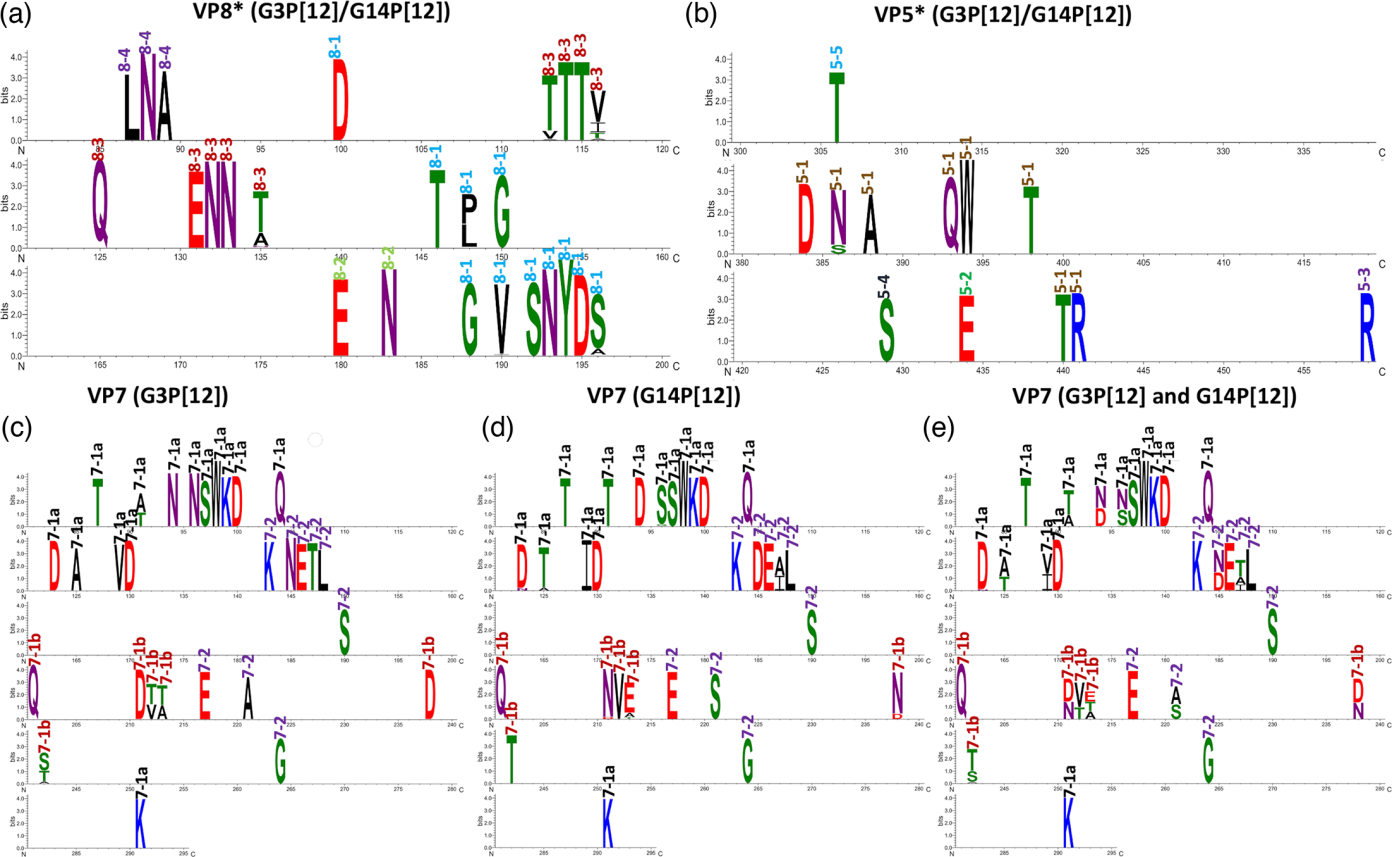
Site-specific sequence conservation in antigenic sites of VP4 and VP7 proteins of ERVA G3P[12] and ERVA G14 P[12]. Site-specific sequence conservation was visualized using Sequence logos generated using Weblogo 3 (https://weblogo.threeplusone.com/). Sequence conservation of antigenic sites of VP8* (**a**) and VP5* (**b**) protein belonging P[12] genotype of ERVA. Similarly, sequence conservation in antigenic sites of VP7 protein is demonstrated for ERVA G3P[12] genotype (**c**) and ERVA G14P[12] genotype (**d**) and for the combination of both ERVA G3P[12] and G14P[12] genotypes (**e**). Height of amino acid symbol indicates the proportion of individual residues at specific position. Amino acids are colour coded based on chemistry, i.e. polar (green), neutral (purple), basic (blue), acidic (red) and hydrophobic (black) (see ‘Methods’ for details). Antigenic sites in VP4 or VP7 protein were labelled in different colour schemes.

Sequence logos were used to visualize sequence conservation in VP4 and VP7 antigenic sites. VP4-based sequence logo (Fig. 7b) for all ERVA G3P[12] and G14P[12] strain sequences showed that antigenic sites in VP5* protein were conserved compared with antigenic sites in the VP8* protein (Fig. 7a). Only single polymorphism was observed in VP5*-based antigenic site 5-1 where 386 position (all numbering for VP5* and VP8* antigenic sites based on the full-length VP4) showed a mixture of asparagine (N) and serine (S) (Fig. 7b). This substitution is a conservative change as N and S are polar amino acids with similar properties. In the VP8* protein, antigenic sites 8-2 and 8-4 were relatively conserved, while 8-1 and 8-3 sites exhibited some variations (Fig. 7a). In antigenic epitope 8-1, polymorphisms were observed at position 148 [proline (P) or leucine (L)) and 196 (serine (S) or alanine (A)]. Similarly in antigenic epitope 8-3, genetic variations were also observed in position 113 [threonine (T) or valine (V)], position 116 [valine (V) or isoleucine (I) or threonine (T) or alanine (A)] and position 135 [threonine (T) or alanine (A)] (Fig. 7a).

Sequence logos were also generated for the VP7 protein for equine G3 (Fig. 7c), G14 (Fig. 7d) and a combination of G3/G14 (Fig. 7e) sequences. Antigenic epitopes 7-1a and 7-2 for G3 were relatively conserved with polymorphisms observed in position 91 (alanine or threonine) for 7-1a (Fig. 7c) and positions 211 (threonine or valine), 212 (threonine or a) and 242 (serine or threonine or alanine) for 7-1b. In contrast, all three antigenic epitopes for G14 showed polymorphisms at various positions (Fig. 7d). Although epitope 7-1a for G-14 showed sequence variations at position 96 (serine or glycine or asparagine), 123 (aspartic acid or asparagine) and 125 (threonine or alanine), these polymorphisms were present only in few viral sequences as indicated by height of the amino acid (Fig. 7d). In antigenic epitope 7-2, G3 showed polymorphism at position 147 (alanine or isoleucine), while the rest of the amino acids residing in 7-2 were conserved. Similarly, epitope 7-1b showed variation at positions 211 (asparagine or aspartic acid), 213 (glutamic acid or alanine or threonine) and 238 (asparagine or aspartic acid) (Fig. 7d).

Combining VP7 sequences from both G3 and G14 genotypes revealed multiple polymorphisms at positions 91, 94, 96, 123, 125, 129, 145, 147, 212, 213, 221, 238 and 242, encompassing three antigenic sites (7-1a, 7-1b and 7-2) (Fig. 7e). This comparative analysis revealed some key genetic polymorphisms that distinguish G3 from G14 strains in VP7-residing antigenic sites, which may hold some clues for antigenic differences between these two genotypes. Inter-genotypic polymorphisms between G3 and G14 are in positions 94 (asparagine or aspartic acid), 96 (asparagine or serine) and 129 (valine or isoleucine) of antigenic site 7-1a as well as in positions 145 (asparagine or aspartic acid) and 221 (alanine or serine) of 7-2 (Fig. 7d). For 7-1b, antigenic sites showed lower sequence conservation when compared with antigenic epitopes 7-1a and 7-2. For 7-1b, positions 213 exhibited genetic polymorphisms in both G3 and G14 genotype. Furthermore, positions 91, 212 and 242 also demonstrated genetic variations in VP7, but these polymorphisms only occurred in G3, not in G14 strains. Some of antigenic epitope residues in 7-1a and 7-2 are completely conserved among G3 and G14 strains analysed. For example, invariant amino acid residues were observed at positions 87, 97–100, 105, 130 and 291 of the antigenic site 7-1a; positions 143, 146, 148, 190, 217 and 264 of antigenic site 7-2; and position 201 of antigenic site 7-1b in both G3 and G14 genotypes. Overall, equine G14 strains appeared to exhibit higher intra-genotypic sequence variations than G3 strains, which probably indicates fitness competition against the G3 genotype for adaptation in host species.

## Discussion

Approximately 16 500 Thoroughbred mares are bred to Kentucky stallions annually, with more than 8000 foals are registered annually as Kentucky bred (foaled in Kentucky). The registered Kentucky bred foal crop alone represents nearly 50% of the Thoroughbred foal crop in North America [9,63]. Despite vaccination with a monovalent inactivated G3P[12] vaccine, foal diarrhoea outbreaks occur, predominantly in the 60–120 days age range [35,64]. More recently, species B RV has also been identified as disease-associated pathogen in neonatal foal diarrhoea outbreaks from Kentucky [65], and other states, thus further emphasizing a need for surveillance of circulating RV strains. One of the bottlenecks to successful adaptation of animal RV *in vitro* is the host immune response. RV replication is restricted in a host species-specific manner, primarily driven by the host innate immune response, and successful RV replication requires overcoming this barrier [66,67]. In this study, we successfully isolated both G3P[12] and G14P[12] with minimal passaging and without introduction of any adaptive changes by comparing the sequences of virus isolates in cell culture to those derived from faecal samples that derived these virus strains. Previous attempts to successfully isolate ERVA G3P[12] were futile, while G14P[12] was isolated after ten blind passages in human colon carcinoma cells (Caco-2) cells, followed by multiple passages in MA-104 cells [35]. In this study, we used genetically engineered MA-104N*V cells defective in IFN and STAT1 response [46,47]. These cells have been used to successfully rescue animal and human RV strains that were previously difficult to rescue using entirely plasmid-based RGS [47]. We successfully isolated ERVA G3P[12] and G14P[12] using this cell line with minimal passaging, and CPEs were observed after the second passage. Defective STAT1 and IFN responses in this cell make them permissive to viral replication to higher titres. It has been shown that STAT1 knockout mice are more permissive to heterologous simian RV than wild-type mice [68]. Thus, this genetically modified cell line may be helpful in successful isolation of difficult to culture RVs. As shown in this study, isolated ERVA G3P[12] and G14P[12] strains replicated robustly in MA-104 cell line, even at low MOI (multiplicity of infection).

Whole genome-based genotyping has become a popular tool in the identification and characterization of the genetic constellation for RVs in general and species A RVs in particular. Using this approach, we found that RVA/Horse-tc/USA/KY-0809N/2021/G3P[12] and RVA/Horse-tc/USA/KY-0316FCL/2021/G14P[12] possessed the highly conserved GCs, but these two genotypes diverged in the middle-layer capsid protein VP6 sequence, with our G3P[12] having I6 and G14P[12] acquiring I2 over the course of their evolution. Such co-segregation of VP7 with VP6 has been previously reported, with G3 associated with I6 while G14 associated with I2 [35,69, 70]. RV anti-VP6 secretory IgA and IgG antibodies are reported to contribute to protection against RV, and thus, the sequence differences in the VP6 protein between RVA/Horse-tc/USA/KY-0809N/2021/G3P[12] and RVA/Horse-tc/USA/KY-0316FCL/2021/G14P[12] may further substantiate the antigenic difference observed between these two dominant genotypes co-circulating in the global equine populations [59,71, 72].

Further analysis of the P genotype-defining VP4 protein sequence showed that RVA/Horse-tc/USA/KY-0809N/2021/G3P[12] and RVA/Horse-tc/USA/KY-0316FCL/2021/G14P[12] appeared to be more distantly related in the VP4 sequence coding for the receptor-binding domain VP8* and the membrane penetration domain VP5*, despite both of them belonging to the P[12] genotype. The previous VP8*-based phylogenetic study also indicated that ERVA G3P[12] and G14P[12] from central Kentucky clustered separately and formed three distinct groups [35]. While VP7-based phylogenetic analysis showed distinct clustering of ERVA G14P[12] strains and G3P[12] strains, the intragenotypic sequence variations were also observed, especially in the ERVA G3 genotype. The G3 genotype has been thought to exhibit the highest degree of the intragenotypic diversity and shows a broad host range and tropism [3]. For example, despite early work showing that ERVA G3P[3] from Argentina might originate from canine/feline [60], recently emerged data indicate that ERVA G3P[3] from Argentina and more recently from India more likely to have originated from bats [30,45]. A new donkey G3P[12] strain from China also formed a cluster with RVA/Bat/G3P[3], indicating that these equine and donkey G3 RVs likely are originated from bats or share the common immediate ancestor(s) with bat G3 viruses. Bat are known reservoir for numerous virus species that infect mammals, including RVA [73,74]. In light of the recent emergence of equine-like G3P[8] RVA in humans, the role of equines in the zoonotic transmission of RVA to humans cannot be neglected. Moreover, the close association between equine G3P[3] and bat G3P[3] also adds to the speculation that equines may play a potential role in the transmission of new RV strains to humans.

Current ERVA vaccination in use is an inactivated monovalent vaccine containing a G3P[12] strain HO-5. ERVA G3P[12]- or ERVA G14P[12]-specific rabbit antisera demonstrated that despite both the inactivated viruses inducing strong immune response against homologous strains, the immune responses in rabbits were sub-optimal for heterologous strains. Field studies in equines have indicated incomplete protection by the current monovalent RV vaccine, as well as sub-optimal heterotypic immune response elicited by the current immunization regime [16,35, 43]. Several amino acid differences, either in antigenic sites or close to antigenic sites, were identified in the immunogenic VP7 and VP4 proteins between ERVA G3P[12] and G14P[12]. These antigenic mismatches may have a profound impact on immunogenicity, which needs further investigations towards developing more effective vaccines that have the cross-protective potency against different genotypes. Sequence conservation analysis showed that antigenic epitopes in VP5* protein are highly conserved. The antigenic epitope in VP5* resides in β-barrel domain of VP4 protein [75]. Engagement of hydrophobic loops in β-barrel domain of VP5* with membrane bilayer is essential for viral penetration into the cellular endosome membrane for releasing double-layered particle into cytosol [75]. B-cell isolated from natural human infection shows neutralizing antibodies directed to the VP5* region [52]. It can envision that VP5*-based sub-unit vaccine may provide better protection against multiple ERVA genotypes, which warrant further investigation. In the VP8* protein, antigenic sites 8-2 and 8-4 and adjourning regions were conserved that could be potential T-cell and B-cell epitopes for vaccine design towards a universal vaccine design. In antigenic epitope 8-1, genetic polytheisms differences were observed at position 148 (proline or leucine), and such non-conservative amino acid in this position may compromise the utility of this epitope as a vaccine candidate [76]. VP8*-based vaccines have been tested in humans as well as in animal models. Despite the failure of a human RVA VP8* subunit vaccine in that the VP8* vaccine showed no superior to a licensed oral RVA vaccine [77], a recent study demonstrated that mRNA-based VP8* nanoparticle vaccines against RV are highly immunogenic in rodents [78], indicating that mRNA vaccine platform may stimulate high-quality immune response over subunit vaccines, which should be explored further in clinical trials. Since VP7 antigenic epitopes contain multiple genetic changes between equine G3 and G14, it can be envisioned that a bivalent vaccine targeting both genotypes may provide better protection against equine RV infections.

ERVA G3P[12] and G14P[12] segregate into two distinct genotypes based on the VP6 protein sequence, exhibiting numerous amino acid differences, including those in the VP6:VP7 protein interaction interface [70]. Antibodies targeting VP6 protein provides protection against disease by inactivating RV post-entry [71,79]. This genotypic difference in VP6 could add up to further limit heterotypic cross-protection by the inactivated G3 vaccine against ERVA G14P[12]. Lastly, it should be noted that the antigenic differences observed between ERVA G3P[12] and G14P[12] was based on rabbit polyclonal antibodies due to lacking genotype-specific antisera made in horses. Further study is needed to further validate this observation using equine anti-ERVA genotype antibodies.

## Conclusion

Despite vaccination, foal diarrhoea outbreaks due to equine RVA remain a significant problem in all horse breeding areas around the globe. Continuous surveillance is crucial to track circulating strains and update vaccination strategies. In this study, we successfully isolated and characterized two prevalent genotypes ERVA G3P[12] and ERVA G14P[12], circulating in the USA. The identified ERVA genotypes show distinct VP6 and VP7 genotypes and amino acid differences in VP7 and VP4 antigenic sites, suggesting limited cross-protection by the current monovalent G3P[12] vaccine. This underscores the need for developing broader-spectrum vaccines against different genotypes or strains of ERVA. The close association between equine G3P[3] and bat G3P[3] raises concerns about the potential role of horses in transmitting new RVA strains to humans. Further research is needed to understand this possibility and to investigate the potential role of horses in zoonotic RVA transmission, as well as development of vaccine with cross-protection against both ERVA G3P[12] and ERVA G14P[12] genotypes.

## Abbreviations

BBD: β-barrel domain
CPE: cytopathic effect
DMEM: Dulbecco’s Modiﬁed Eagle Medium
EM: electron microscopy
ERVA: equine rotavirus species A
FFU: fluorescent foci unit
GC: genome constellation
GMT: geometric mean titres
IFA: immunofluorescence assay
NSP1: non-structural protein 1
RFD: Rossmann fold domain
RT-PCR: reverse transcription-PCR
RV: rotavirus
RVA: rotavirus species A
STAT1: signal transducer activator of transcription 1
